# Characterization, Antimicrobial and Anticancer Properties of Palladium Nanoparticles Biosynthesized Optimally Using Saudi Propolis

**DOI:** 10.3390/nano11102666

**Published:** 2021-10-11

**Authors:** Maged S. Al-Fakeh, Samir Osman Mohammed Osman, Malek Gassoumi, Mokded Rabhi, Mohamed Omer

**Affiliations:** 1 Department of Chemistry, College of Science, Qassim University, Buraidah 51452, Saudi Arabia; m.alfakeh@qu.edu.sa; 2 Taiz University, Taiz 3086, Republic of Yemen; 3 Department of Physics, College of Science, Ibb University, Ibb 009674, Yemen; 4 Engineer College, Aljanad University for Science & Technology, Taiz 009674, Yemen; 5 Department of Physics, College of Science, Qassim University, P.O. 64, Buraidah 51452, Saudi Arabia; malek.gassoumi@univ-lille1.fr; 6 Unite de Recherche Matériaux Advances et Nanotechnologies, Institut Supérieur des Sciences Appliquées et de Technologie de Kasserine, Université de Kairouan, BP 471, 1200 Kasserine, Tunisia; 7 Department of Plant Production and Protection, College of Agriculture and Veterinary Medicine, Qassim University, Qassim 51452, Saudi Arabia; mokded.rabhi@gmail.com; 8 Department of Radiologic Science, College of Applied Medical Sciences, Buraidah 51452, Saudi Arabia; alkajm@gmail.com

**Keywords:** Pd-NPs, antimicrobial, anticancer

## Abstract

Due to their unique physicochemical characteristics, palladium nanoparticles (Pd-NPs) have shown tremendous promise in biological applications. The biosynthesis of Pd-NPs employing Saudi propolis has been designed to be environmental, fast, controlled, and cost-effective. The formation and stability of biosynthesized Pd-NPs by Saudi propolis extract were proved by ultraviolet–visible spectrophotometry (UV-Vis), Fourier-transform infrared spectroscopy (FT-IR), and Zeta potential analysis. Transmission electron microscopy (TEM), scanning electron microscopy (SEM), and X-ray diffraction (XRD) findings show that the average particle size of Pd-NPs is between 3.14 and 4.62 nm, which is in quantum scale. The Saudi propolis enhanced the antimicrobial activity against *B. subtilis*, *S. aureus*, *E. coli*, *K. pneumoniae*, and *C. albicans*. Pd-NPs show effective anticancer activity against ductal carcinoma (MCF-7) with IC50 of 104.79 µg/mL.

## 1. Introduction

**Scheme 1:**
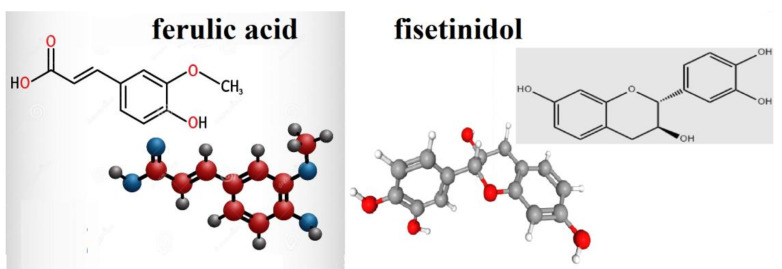
Ferulic acid and fisetinidol molecular structure.

Propolis has attracted researchers’ attention due to its wide application in ‘natural’ makeup, medicines, and many other products. Propolis is known as a resinous material produced by bees from buds and exudates of the plants, which is mixed with bee enzymes, pollen, and wax. The word ‘propolis’ is a Greek word containing two parts; the first part ‘pro’ means ‘in front’, and the second part ‘polis’ means ‘the city’. So, the whole word means ‘defense of the hive’. Different propolis samples from varied botanic and geographic sources were studied. It was found that substantial changes occur in some chemical components of propolis (polyphenols and flavonoids) [1]. The synergistic action of the two active ingredients, ferulic acid and fisetinidol (in Scheme 1), is thought to be responsible for the bioactivity of Saudi propolis. The findings suggested that Saudi propolis could be used as an antitrypanosomal and anticancer agent in cancer treatment [2].

Since the turn of the century, nanotechnology research has grown in popularity and utility. At the nanoscale, nanotechnology has produced a variety of materials. Pharmaceuticals associated with microorganisms, building polymers withstanding mechanical stress, and surface protection paints are all examples of nanotechnology products in the chemical industry [3,4].

Nanomaterials are a broad category of materials that contain particulate substances with a minimum dimension of 100 nanometers. Researchers discovered that the size of a substance would affect its physicochemical properties, emphasizing the significance of these materials [5].

Palladium belongs to the periodic table’s column 10; however, its outer electron shell structure (Pd = 4d10, 5s0) is quite unique compared to other members of the group. It is the least dense and has the lowest melting point of all the platinum group metals (Ru, Rh, Pd, Os, Ir, Pt). Palladium nanoparticles (Pd-NPs) have a variety of exceptional characteristics, including excellent catalytic, optical, mechanical, physical, and electrical properties, as well as size and shape variety. Pd-NPs have been shown to have great potential in photothermal, photoacoustic, and antimicrobial/antitumor applications and as gene/drug carriers, biosensors, and prodrug activators in preliminary studies [6,7,8,9].

Because of their unique physicochemical features, palladium nanoparticles (Pd-NPs) have shown considerable promise in biomedical applications. Pd-NPs have been synthesized using a variety of traditional physical and chemical processes. These approaches, on the other hand, may include the use of hazardous chemicals and reaction conditions that are potentially harmful to human health and the environment. As a result, environmentally acceptable, quick, and cost-effective techniques for the synthesis of Pd-NPs have been established. Pd-NPs were made from bacteria, seaweeds, yeast, fungi, plants, and plant extracts [10,11,12,13].

Palladium nanoparticles have been shown to have antibacterial properties that are size-dependent. It was found that Pd-NPs are more toxic than Pd^2+^ ions against Gram-positive *S. aureus* bacteria. Pd nanoparticles with a smaller diameter, such as 2 nm, were found to be more toxic than those with larger diameters, such as 2.5 or 3.1 nm [14,15]. For average diameters of 200, 220, and 250 nm, the green synthesis and characterization of Pd-NPs employing a quercetin-derived flavonoid showed the strongest antifungal activity for both fungi examined [16].

Tea-biosynthesized palladium nanoparticles (Pd-NPs) have been identified as drug carriers, ranging in size from 6 to 18 nm. These palladium nanoparticles have strong scavenging properties, antimicrobial activity, and cytotoxic characteristics in human leukemia cells while having no negative effects on normal human fibroblast cells [17].

Pd-NPs entrapped inside a modular polymeric framework were employed as a prodrug activation approach in cultured cells to synthesize an anticancer agent from two nontoxic precursors, causing cell death [18].

At a dosage of 25 mg/mL, Pd-NPs made from the fruit extract of *Couroupita guianensis* Aubl. showed impressive antibacterial activity against both Gram-positive and Gram-negative bacterial pathogens. Furthermore, the anticancer potential of these Pd-NPs was notable in human lung carcinoma cells, with 50% mortality at 121 mg/mL, and the blood compatibility of synthesized Pd-NPs shows that it does not interfere with whole RBCs [19].

There is no unique mechanism of Pd-NP biosynthesis that has been adopted, but there are hypotheses. Four essential bottom-up processes were postulated to explain the mechanism of biosynthesized nanoparticle production [11]. The bioreduction and nucleation of metal ions are the initial activation step in the biosynthesis of Pd-NPs. In the second process, the tiny Pd-NPs are grown and agglomerated and become larger and more thermodynamically stable [20]. In the third step, depending on the optimum circumstances, a range of Pd-NPs forms are produced. A plant extract that contains a variety of functional groups serves as a capping agent and is utilized to keep the Pd-NPs stable throughout this step [21].

Bacteria, plants, algae, fungi, and yeast are all used in the production of nanoparticles. In the biosynthesis of Pd-NPs using plants, biomolecules are thought to act as reducers, whereas heterocyclic chemicals act as capping agents. Various studies have linked polysaccharides and other organic and water-soluble substances to metallic ion reduction and the stability of nanoparticles [22,23].

**Scheme 2:**
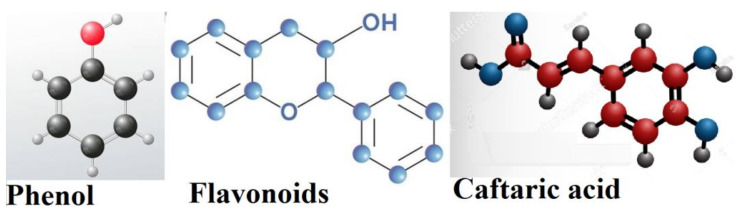
Some polyphenolic compounds in propolis.

Propolis is a plant-mediated nanoparticle formation, and the main active compounds in propolis are polyphenolic phytochemicals found in many plants and fruits, such as flavonoids, phenolic acids, tannins, and stilbenes [24,25,26,27]. Some common polyphenolic compounds are shown in Scheme 2. Palladium bioreduction may have been aided by the hydroxyl functional groups found in polyphenols [28].

This study aims to biologically prepare nanomaterials (nano-palladium) that have better antibacterial, viral, and anticancer properties than those found in previous studies in this area. The novelty of this study is that it takes advantage of the unique characteristics of Saudi propolis, which differs from propolis from other regions in the world due to its geographical location and bee food. The characteristics of Pd-NPs developed using the Saudi propolis extract were tested in addition to combining the extract of this Saudi propolis in an optimized way with palladium dichloride to obtain the best method for biological preparation of Pd-NPs.

## 2. Materials and Methods

### 2.1. Materials

Al-Qassim Saudi commercial propolis was gathered from the market. The palladium dichloride was supplied by Loba Chemie (Mumbai, India). Sodium hydroxide (NaOH) and hydrochloric acid (HCl) were provided by Sigma Aldrich Chemicals (Darmstadt, Germany). The deionized water and ethanol were of analytical quality.

### 2.2. Propolis Extract Preparation

To obtain an extract from Saudi propolis, 2 g of propolis granules was mixed with 100 mL deionized water. To obtain a suitable extract, the mixture was agitated and heated to 60 °C for 30 min. The extract was then filtered through Whatman filter paper and stored in a cool place to be used again.

### 2.3. Optimal Biosynthesis of Palladium NPs

Firstly, 0.0355 g PdCl_2_ was dissolved in 100 mL deionized water to make a 2 mM solution. To achieve optimal conditions of Pd nanoparticle preparation, the biological preparation of palladium nanoparticles was carried out with the best values of the ratio between propolis extract and palladium dichloride, pH, reaction time, and temperature. The optimal conditions for biosynthesis Pd-NPs were as follows:

#### 2.3.1. Ratio and Concentration Effects

The reaction mixture was first formed by adding certain volumes of 2 mM PdCl_2_ solution with specific volumes of propolis extract (0.2 g/L) and then stirring for one hour. The ratios in milliliters were 2:8, 4:6, 5:5, 6:4, and 8:2. The color of the mixture was changed, confirming the preparation of Pd-NPs. The next stage was studying the effect of concentrations of both PdCl_2_ and propolis extract. One concentration was kept constant and the other was changed until the optimum concentrations for the mixture were obtained.

#### 2.3.2. Effect of pH

Samples of the mixture (propolis extract and palladium dichloride aqueous solution) were prepared with the best concentrations and ratio achieved from the previous step, but with different pH values (4, 6, 9, and 11).

#### 2.3.3. Temperature Effect

To explore the effect of temperature on the biological preparation of palladium nanoparticles, numerous samples of the mixture (PdCl_2_ and propolis extract) were prepared with the best values of the ratio, concentration, and pH. In these tests, every sample was placed in a water bath that was kept at a constant temperature. The temperatures analyzed were 25, 35, 45, 55, 65, 75, and 85 °C.

#### 2.3.4. Effect of Reaction Time

To determine the optimum reaction time of Pd-NPs biosynthesis, the sample of the mixture was prepared with the optimum values of ratio, concentrations, pH, and temperature. The reaction time for the biosynthesis of palladium nanoparticles was investigated over 70 min.

All experiments above for optimal biosynthesis of Pd-NPs were monitored using a UV-Vis spectrophotometer (Shimadzu Europe, Duisburg, Germany).

### 2.4. Characterization Techniques

Shimadzu Ultraviolet Spectrometer 2700 (Shimadzu Europe, Duisburg, Germany) was used to monitor the biosynthesizing of palladium nanoparticles using Saudi propolis extract with optimal conditions.

To observe the effect of biomolecules on the reduction of Pd nanoparticles and capping of the bioreduced nanoparticles, Nicolet 6700 FTIR spectrometer (Thermo Fisher Scientific, Waltham, MA, USA) was used.

X-ray diffraction analysis of Pd nanoparticle crystalline growth was performed using a Bruker D8 Discover diffractometer (Bruker, MA, USA) equipped with a Cu microfocus X-ray source (0.15406 nm) and a 2-dimensional Vantec 500 detector (Bruker, MA, USA). On a glass slide, a drop of the nanoparticle suspension was deposited. The measurements were taken with a step scan of 0.0052 in a 2-theta range of 5 to 80°. The data analysis was performed using the program DIFFRAC.EVA (Bruker, MA, USA).

Zeta potential was measured using a Nicomp 380 DLS/ZLS (NICOMP, PSS, FL, USA).

TEM examination was executed using a JEOL GEM-1010 transmission electron microscope (JEOL, Ltd., Akishima, Tokyo, Japan) with a 70 kV accelerating voltage to determine the size and shape of Pd-NPs. A drop of a particle-containing liquid was dropped onto a copper grid and kept at room temperature by permitting water to evaporate.

EDX (FEI Quanta FEG 450) (ThermoFisher Scientific, Waltham, MA, USA) was utilized to gather qualitative information on constituents in the product of Pd nanoparticles. A scanning electron microscope (FEI Quanta FEG 450) (ThermoFisher Scientific, Waltham, MA, USA) was used to capture nanostructure and morphology pictures of Pd nanoparticles.

### 2.5. Antimicrobial Experiment Design

*Bacillus subtilis*, *Staph. aureus*, *Escherichia coli*, *K. pneumoniae*, *Candida albicans*, and *Aspergillus niger* were employed to test the antimicrobial activity of palladium nanoparticles. For the antibacterial activity test, 50 mg dried palladium nanoparticles dissolved in 1 mL deionized water was employed. For that purpose, the agar diffusion method was used. The standard antibacterial agent was gentamicin (10 µg/mL). Mueller–Hinton agar plates were sown with the test microorganisms at 1.8 × 10^8^ cfu/mL (0.5 OD^600^). Plates were evaluated for the presence of inhibitory zones after a 24 h incubation period at 37 °C. For this test, 50 mg/mL of palladium nanoparticle solution was used. For measuring the inhibition zones enclosing the wells in millimeters, only halos larger than 6 mm were used. The inhibition zones observed for each experiment are the average of three attempts [29].

### 2.6. Cytotoxicity Assay on MCF-7 Cells

#### 2.6.1. Cell Culture

MCF-7 is a cell line originating from *Homo sapiens*, human. MCF-7 (HTB-22) cell line was supplied by ATCC (American Type Culture Collection, VA, USA) and cultured in RPMI-1640 (Gibco BRL, Grand Island, NY, USA) at 37 °C in a 90% humidified incubator with 5% CO_2_.

#### 2.6.2. MTT Protocol

The cytotoxicity was determined according to the MTT protocol. To develop a complete monolayer sheet, 96-well tissue culture plates were inoculated with 1 × 10^5^ cells/mL (100 µL/well) and incubated at 37 °C for 24 h. Growth medium was decanted from 96-well microtiter plates after a confluent sheet of cells was formed, and the cell monolayer was washed twice with wash media. A 2-fold dilution of the tested sample was made in RPMI medium with 2% serum (maintenance medium). One hundred microliters of each dilution was tested in different wells; 3 wells were left as control, receiving only maintenance medium. The plate was incubated at 37 °C and examined. Cells were checked for any physical signs of toxicity, e.g., partial or complete loss of the monolayer, rounding, shrinkage, or cell granulation. MTT solution was prepared (5 mg/mL in PBS) (Bio Basic Inc., Ontario, Canada). Twenty-microliter MTT solutions were added to each well and then placed on a shaking table, 150 rpm for 5 min, to thoroughly mix the MTT into the media. The cells were incubated (37 °C, 5% CO_2_) for 1–5 h to allow the MTT to be metabolized. The media were then removed from the plate, which was dried with paper towels to remove any remaining residue. Formazan should be resuspended in 200 µL DMSO (MTT metabolic product). To completely dissolve the formazan in the solvent, it was placed on a shaking table at 150 rpm for 5 min. The optical density was observed by microscope at 560 nm after subtracting the background at 620 nm. So, the number of cells should be proportionate to the optical density [30,31,32].

#### 2.6.3. Statistical Analysis

Using Excel, statistics were employed to compare the concentration of biosynthesized palladium nanoparticles to the number of cells. The cytotoxicity tests were performed three times, and the results were calculated using mean values and standard deviation (SD). The P-value was discovered to be less than 0.05, which is considered statistically significant.

## 3. Results

### 3.1. UV Spectrometer Results

**Figure 1:**
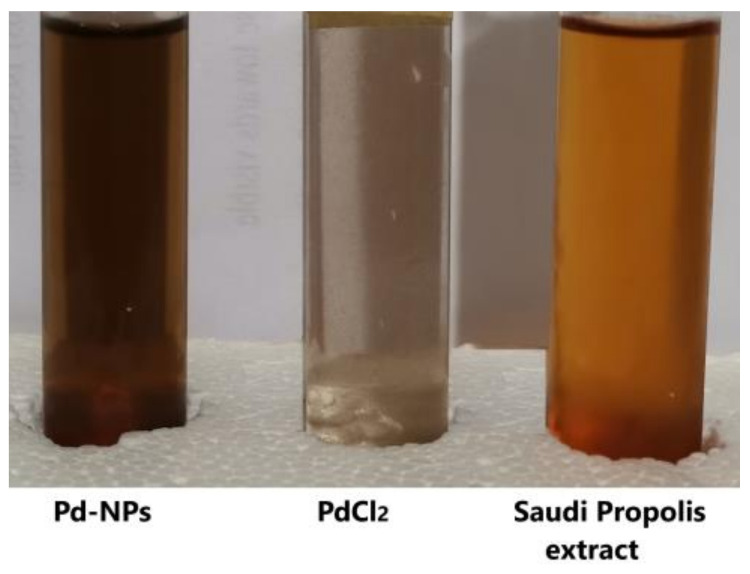
The photographs of the elements that interact with each other and the finished product (Pd-NPs).

For controlling the synthesis and stability of Pd-NPs, the absorption spectra of the synthesized palladium nanoparticles were recorded. As the extract concentration increased, the color of the solutions altered from light yellow to yellowish-black to deep black. The colors of the propolis extract, the aqueous solution of palladium (II) chloride, and the palladium nanoparticle solution are shown in Figure 1.

#### 3.1.1. Concentrations and Ratio Effects

**Figure 2:**
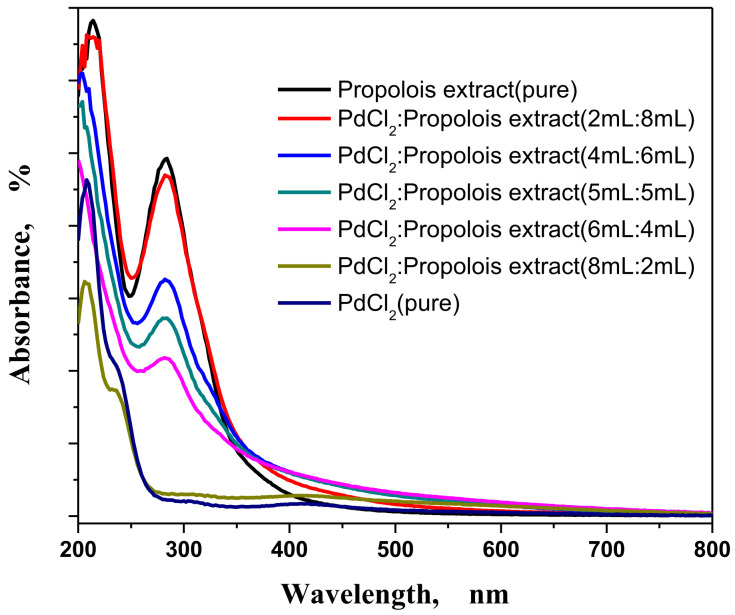
The effect of ratio on biosynthesis of palladium nanoparticles.

**Figure 3:**
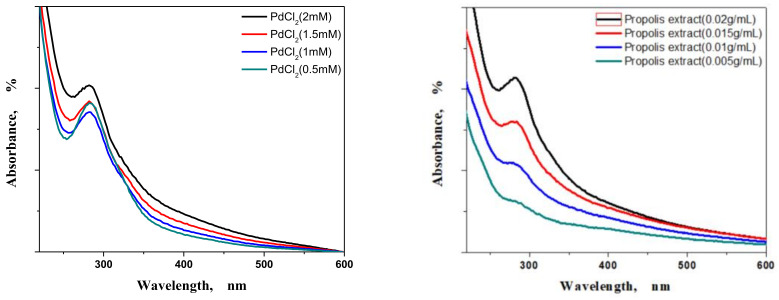
The effects of PdCl_2_ and propolis extract concentrations on palladium nanoparticle biosynthesis.

Figure 2 reveals the confirmation of palladium nanoparticle production compared to pure extract. As shown in Figure 3, the impact of the ratio of palladium dichloride and propolis extract was investigated.

#### 3.1.2. pH Factor

**Figure 4:**
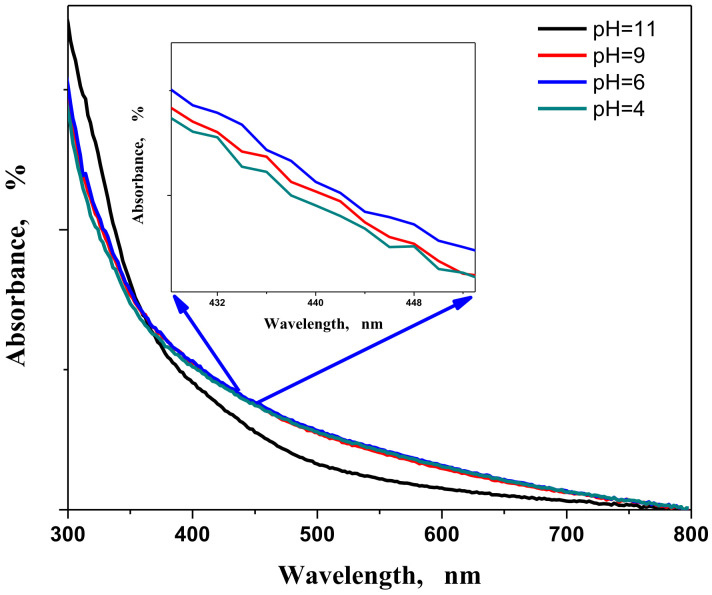
The impact of pH on biosynthesis of palladium NPs.

Figure 4 depicts the impact of pH on the production of palladium NPs. The absorbance of UV rays increases and subsequently decreases as the pH climbs from 4 to 11. The highest value of absorbance was at pH value 6. Furthermore, the Pd nanoparticles’ yellowish-black hue was seen quickly after the PdCl_2_ was combined with the extract.

#### 3.1.3. Temperature Factor

**Figure 5:**
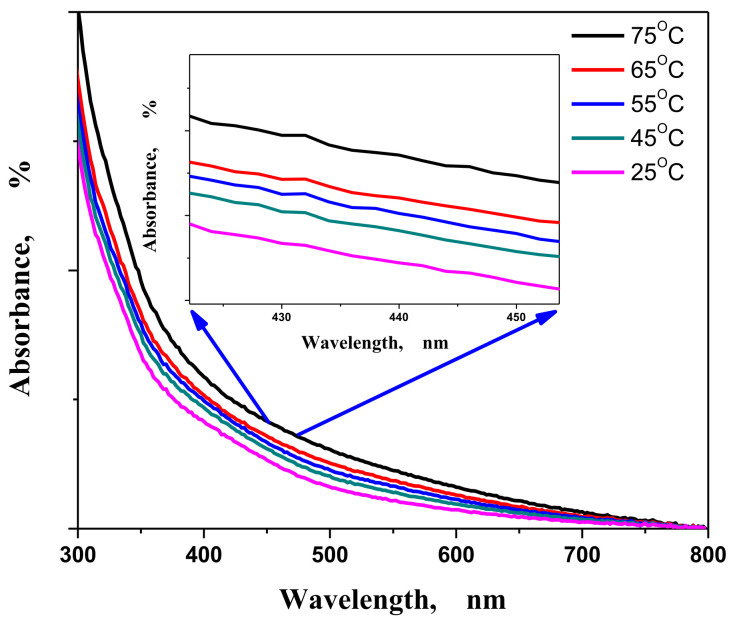
The effect of temperature on biosynthesis of palladium nanoparticles.

The UV–visible spectra of palladium nanoparticles prepared at various temperatures are shown in Figure 5. It can be seen that as the temperature rises, the absorbance rises as well.

#### 3.1.4. Contact Time Effect

**Figure 6:**
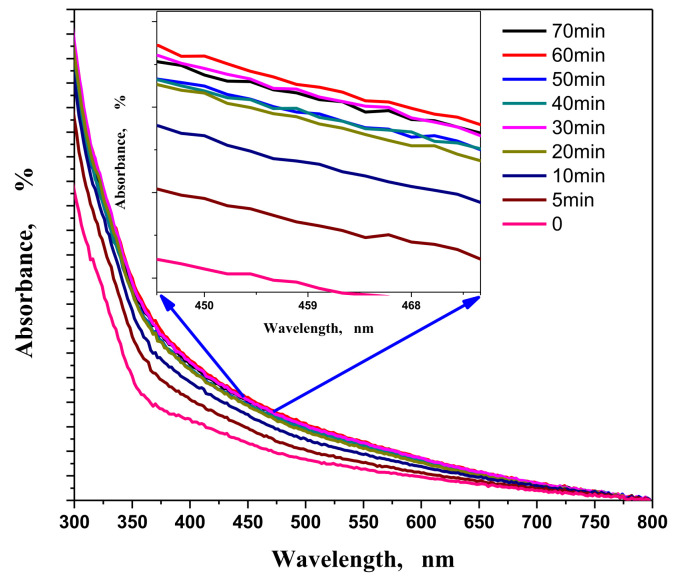
Contact time of the biosynthesis of palladium nanoparticles at room temperature.

Contact time, also known as induction time, is the length of time necessary for nanoparticles to form in solution. As seen in Figure 5, this was demonstrated in several investigations. Figure 6 displays the UV–visible spectra of palladium NPs as a function of time after adding 4 mL of propolis extract to 6 mL PdCl_2_. As the reaction time was extended, the absorbance in the visible range varied nonuniformly, with the maximum absorbance recorded after 60 min at room temperature.

### 3.2. XRD Analysis

**Figure 7:**
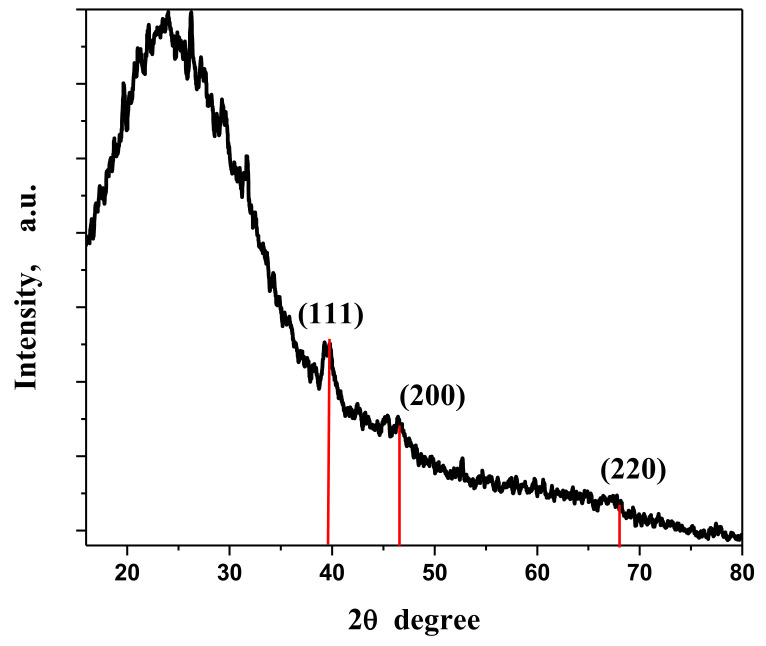
XRD patterns of the biosynthesis of palladium nanoparticles.

To evaluate the size of particles and describe the crystalline structure of the produced Pd-NPs, dried Pd-NPs were analyzed using XRD. Figure 7 shows the XRD patterns of dried Pd nanoparticles synthesized at 25 °C using Saudi propolis extract. Palladium NPs have a face-centered cubic (fcc) structure, according to the XRD findings of Pd/extract [33,34,35]. Peak positions 39.55, 46.42, and 68.1° (as in Figure 7) were related to dried Pd-NPs (JCPDS: 87-0643). The fcc lattice planes of Pd-NPs were (1 1 1), (2 0 0), and (2 2 0) [33,36].

As can be seen from the XRD pattern, palladium nanoparticles made with propolis extract were basically crystalline.

The average nanocrystalline size was estimated employing the Debye–Scherrer method [37], D = λk/βcosθ, where D is the crystal size, k equals unity, λ is the X-ray source wavelength (0.1541 nm), β is the full width at half maximum (FWHM), and θ is the diffraction angle corresponding to the lattice plane (111). Using the Debye–Scherrer equation, the average crystallite size is 4.62 nm.

### 3.3. FT-IR Analysis

**Figure 8:**
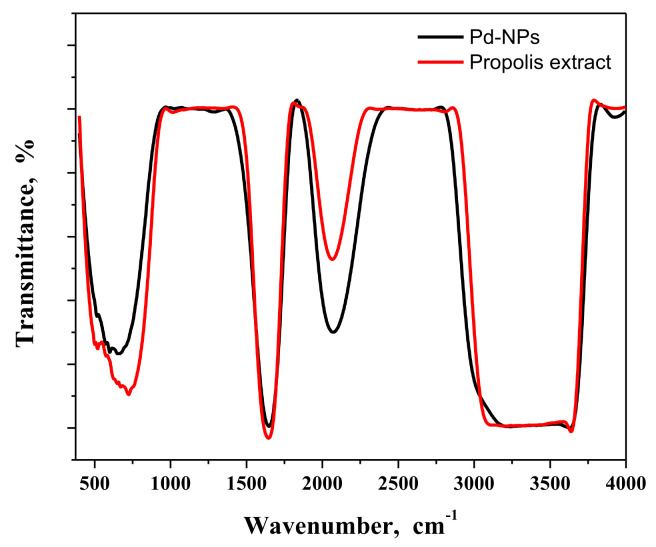
FT-IR spectra of propolis extract and palladium nanoparticles.

The propolis extracts’ FT-IR peaks (Figure 8) reveal strong and broad absorbance peaks in the 3770–2870 cm^−1^, 2300–1900 cm^−1^, 1750–1500 cm^−1^, and 900–350 cm^−1^ areas.

There are other small faint peaks in the 4000–3800 cm^−1^ and 1400–1000 cm^−1^ areas. It is noted that this band becomes stronger and broader in Pd-NPs. There is a strong peak in the 1640 cm^−1^ area.

### 3.4. Zeta Potential

**Figure 9:**
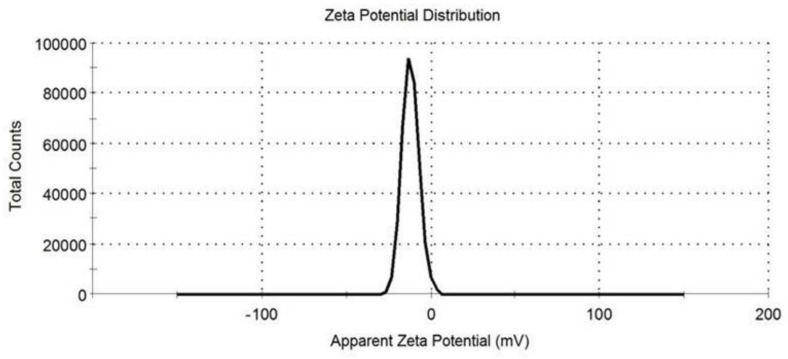
The zeta potential of biosynthesized palladium nanoparticles.

The zeta potential of palladium nanoparticles biosynthesized using Saudi propolis extract was estimated. The value of zeta potential is −9.2 mV (as in Figure 9).

### 3.5. TEM, SEM, and EDX Measurements

**Figure 10:**
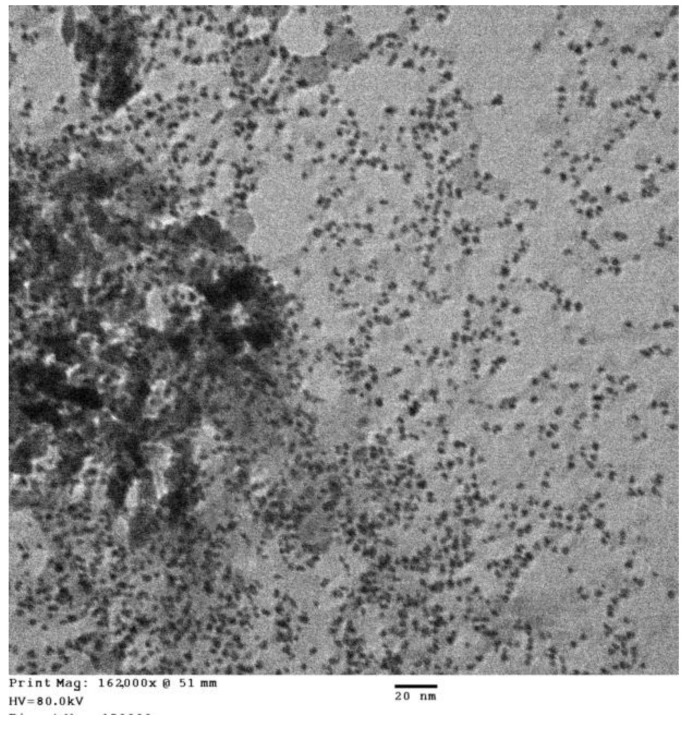
TEM image of biosynthesized palladium nanoparticles.

Figure 10 shows a typical TEM picture of palladium nanoparticles biosynthesized from Saudi propolis. The average particle size is 3.14 nm.

**Figure 11:**
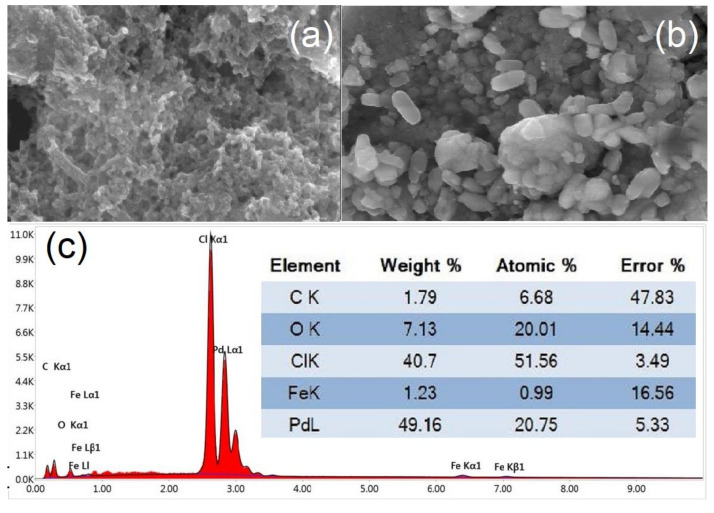
The images of SEM of Pd-NPs are shown in (**a**,**b**). (**c**) EDX data of biosynthesized palladium nanoparticles.

The morphology of palladium nanoparticles produced using Saudi propolis extract at various concentrations and states was examined using a scanning electron microscope (Figure 11a,b). Figure 11a is an as-prepared sample. Figure 11b is the dried Pd-NP sample.

The energy dispersive X-ray fluorescence spectrometry (EDX) spectrum of Pd-NPs was obtained (Figure 11c).

### 3.6. Antimicrobial Activity of Palladium Nanoparticles

**Figure 12:**
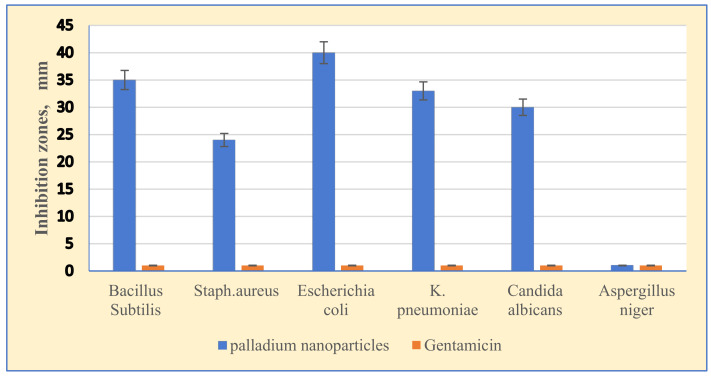
Inhibitory effect of biosynthesized palladium nanoparticles using Saudi propolis on different microbes (inhibition zones in mm).

The Pd-NPs’ antimicrobial activity against *S. aureus*, *B. subtilis*, *K. pneumoniae*, *E. coli*, and *Candida albicans* is shown in Figure 12. Palladium nanoparticles had no effect on *Aspergillus niger*. Palladium nanoparticles had the greatest antibacterial effectiveness against *Escherichia coli*, *Bacillus subtilis*, *K. pneumoniae*, and *Candida albicans* according to the findings in Figure 12. Pd-NPs show a moderate effect against *Staph. aureus*. As shown in Figure 12, palladium nanoparticles, prepared as dried sample, have no effect on *Aspergillus niger*.

### 3.7. In Vitro Anticancer Activity of Palladium Nanoparticles against MCF-7 Cell Line

**Figure 13:**
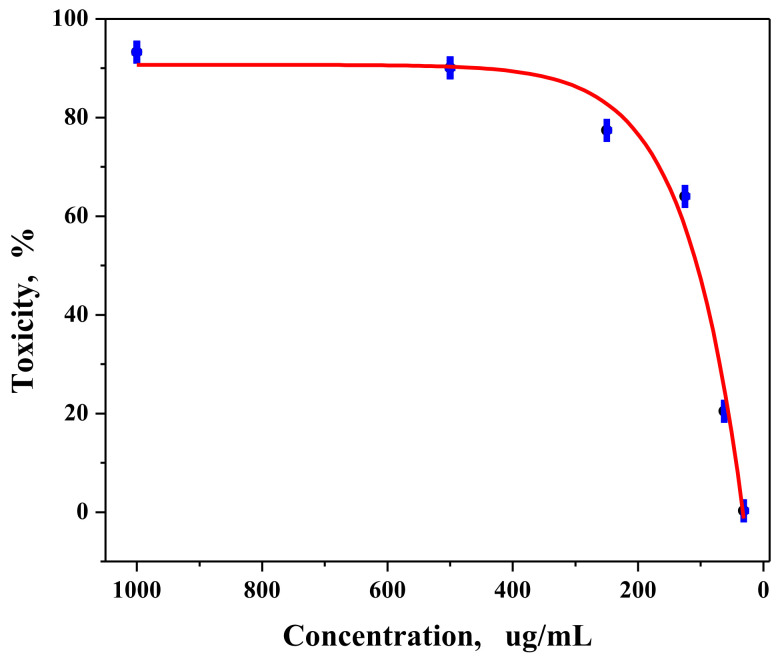
The toxicity of palladium nanoparticles on MCF-7 cells at various doses.

**Figure 14:**
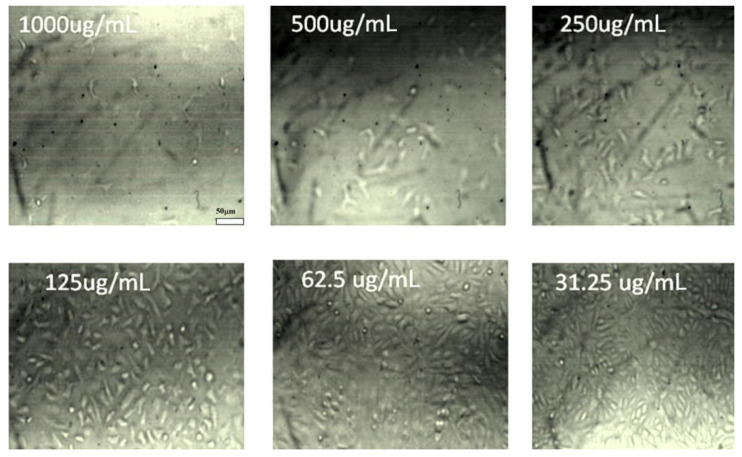
The impact of palladium nanoparticles at various doses on MCF-7 cells.

The in vitro MTT assay was employed to test the cell viability of biosynthesized palladium nanoparticles. This MTT test was executed on MCF-7 cell lines of breast cancer at various dosages. Biogenic Pd-NPs were placed in MCF-7 cells at various doses (1000–31.25 g/mL) for one day, and percent inhibition of cell growth was measured by the MTT test. The viability of the cells decreased significantly when the dosage concentration was increased from 31.25 to 1000 g/mL. Dose–response curve (DRC) was used to measure the concentration that inhibits cell growth by 50% (IC50). The Origin software was used to determine the IC50 value (Figure 13). The IC50 value of Pd-NPs for the MCF-7 cell line was calculated to be 104.79 µg/mL using statistical analysis. The microscopic images of MCF-7 cells were taken at different concentrations of palladium nanoparticles, as shown in Figure 14. The toxicity impact of palladium nanoparticles at various doses on MCF-7 cells is illustrated in Figure 14.

## 4. Discussion

### 4.1. Characterization of Pd-NPS

The change in the color of the solutions from light yellow to yellowish-black to deep black suggests the creation of palladium nanoparticles since the change in color was induced by surface plasmon vibration excitation in the palladium nanoparticles, as the color of the final output in Figure 1.

The formation of Pd-NPs was monitored with increasing concentration of both PdCl_2_ and propolis extract, as shown in UV-Vis spectra (Figure 3). These findings are consistent with prior publications on palladium nanoparticle production [11,17,38].

The optimum value of absorbance of the mixture was at pH value 6. It is noticed that Pd nanoparticles made with propolis extract as a reducing agent are generally stable in solution and evenly distributed. The rise in absorbance is due to an increase in particle size because of the negative surface charges; the functional groups serve as a capping agent, preventing particle aggregation [39,40]. The pH effect is easy to attribute to the variation in nanoparticle stability. During the particle production process, the pH of the electrolyte plays a significant role [41]. This leads to the conclusion that the size of nanoparticles is decreasing, which may be due to decreased particle aggregation due to full charging of the clusters, which maximized repulsive electrostatic interactions [42].

The optimum temperature of formation Pd-NPs was 75 °C, as shown in Figure 5. This finding demonstrated that raising the temperature of the mixture will accelerate the palladium NP production as compared to room temperature. It is proposed that the absolute negative charge grew as the temperature rose, indicating that greater temperatures produced more nanoparticles [40].

Contact time, also known as induction time, is the length of time necessary for nanoparticles to form in solution. Its value is determined by the techniques used to detect particle formation in solution, the reaction’s agitation speed, and the presence of contaminants in the reaction solution [43]. Heating energy is widely recognized for hastening the reaction of a combination. As seen in Figure 5, the optimum reaction time was 30 min.

The reducing agents in propolis extract converted the Pd(II) ions in the PdCl_2_ solution to zero-valence Pd atoms. Reduced Pd atoms attempted to cluster together, and tiny particles tried to combine into big particles to lower total surface energy, eventually resulting in the development of larger Pd particles. The organic long chains inhibited the aggregation of nanoscale Pd particles, preventing further growth of the Pd particles. This leads to obtaining the crystalline and amorphous structure of Pd-nanoparticles [44]. Palladium NPs have a face-centered cubic (fcc) structure, according to the XRD findings of Pd/extract [33,34,35]. The fcc lattice planes were (1 1 1), (2 0 0), and (2 2 0) [33,36]. The influence of the nanosized particles caused a broadening of the diffraction peaks, and the transition elements in propolis extract caused some peaks to be shown [45]. The average nanocrystalline size of Pd-NPs was 4.62 nm. This value is in agreement with TEM measurements.

The existence of flavonoids, which cause the natural color of propolis powder and liquid extract, is indicated by peaks in the 2300–1900 cm^−1^ range in FTIR, as shown in Figure 8. It is noted that this band becomes stronger and broader in Pd-NPs. Palladium ions are reduced to Pd-NPs by biomolecules found in the extract of propolis, such as terpenoids, flavonoids, and phenolic substances. The mechanistic function of flavonoids as a reducer and capping agent in the production of metal NPs has been described [46]. The largest functional peak, at 3430 cm^−1^, displays the presence of phenolic hydroxyl groups in the structure, which practically confirms the existence of friedelin, lupeol, and β-sitosterol. The strong peak in the area 1640 cm^−1^ relates to the aromatic stretching of C–H, which is linked to the phenolic ring structure. The presence of such groups in the aromatic ring structure is indicated by the unsaturated C=C structure, as demonstrated by the peak in the 2065 cm^−1^ area [17].

The electrochemical equilibrium at the particle–liquid interface is measured by the zeta potential. It is one of the primary factors known to impact colloidal particle stability since it quantifies the degree of electrostatic repulsion/attraction between particles. It is worth noting that when it comes to nanoparticle dispersions, the term ‘stability’ refers to the dispersion’s resistance to change over time [40,47]. The value of zeta potential shows the potential stability of the Pd nanoparticle solution, and the nanoparticles’ surfaces are negatively charged (−9.2 mV) and evenly dispersed throughout the medium. According to published research, particles in a stable suspension have zeta potential ranging between +30 and −30 mV [48,49].

According to the TEM image, the palladium nanoparticles have a variety of sizes and forms. The average particle size was determined to be 3.14 nm, which matched the XRD results. These sizes of particles are within the scale of quantum dots.

The morphology of palladium nanoparticles forms nonuniform shapes. In Figure 11a, in the as-prepared sample, the particles are in cluster shapes with smaller sizes (at low concentration and in dried liquid state). In Figure 11b, the particles are in cylindrical/capsule shape shapes with larger sizes (at high concentration and in solid state). It is clear that increasing the concentration of the mixture will increase the size of nanoparticles due to the aggregation of more ions (Pd^+^) and functional groups (from propolis extract).

The components contained in the sample were clearly identified using energy dispersive X-ray fluorescence spectrometry (EDX) (Figure 11c). The presence of Pd-NPs was verified by the observation of a Pd l-shell emission peak at 5.5 keV (kα). At an energy of 1.1 keV and below, certain faint signals from C, O, and Fe were detected, which are attributable to metal ions present in Saudi propolis extract. The element Cl, on the other hand, produced a strong signal at 11 keV, which was caused by the PdCl_2_ compound. Palladium has the greatest value (49.16%) and can be categorized accurately based on the elemental weights in the nanoparticles.

### 4.2. Antimicrobial and Anticancer Mechanisms

The key driver of palladium nanoparticles’ antibacterial and anticancer activities is still to be recognized, but it can be connected to the mechanical action of Pd^+^ ions against microbes, bacteria, and cancer, in which the aggregation of Pd-NPs from the aqueous solution significantly contributes to the saturation of the cell’s enzymes and proteins [12]. Palladium nanoparticles are thought to have three antibacterial and anticancer processes. The first mechanism is that nanoscale palladium particles stick to the cell wall and hinder cell/bacterial development and multiplication, causing the cell wall to be disrupted and unable to protect the cell’s interior [50]. The second mechanism is that Pd-NPs penetrate the bacterial cell and alter the normal development of nucleic acids, which leads to cell death or at the very least DNA damage [49]. The third mechanism is that the bacterial cell wall is irreversibly disrupted by the engagement of Pd^+^ ions with sulfur-containing proteins in the cell wall. When testing antimicrobial activity, this suggested mechanism was also concluded as the primary antimicrobial mechanism [51,52,53,54].

Palladium nanoparticles’ antimicrobial activity is influenced by a number of factors, including particle shape, size, morphology, and surface charge. Because of the structure of the bacterial cell wall, nanoparticles can enter the nuclei of bacteria, inactivating DNA and enzymes and causing cellular death, particularly in Gram-negative bacteria [55,56].

#### 4.2.1. Antimicrobial Activity of Palladium Nanoparticles

Research surveys showed that both palladium nanoparticles and propolis have antimicrobial and anticancer properties [7,57,58]. When compared to prior studies, the data show that biosynthesized palladium nanoparticles combined with Saudi propolis had higher antibacterial action against these microorganisms [59]. This enhances the effect of Saudi propolis in increasing the antimicrobial activity of palladium NPs.

#### 4.2.2. Anticancer Activity of Palladium Nanoparticles against MCF-7 Cell Line

Several research papers proved the effective anticancer activity of Pd nanoparticles [12,60]. This implies that the synthesized palladium NPs with Saudi propolis demonstrated greater efficacy in obtaining reduced cell viability or increased toxicity. As a result, Saudi propolis extract with palladium nanoparticles plays a critical function in enhancing cytotoxicity. It is noted that the IC50 is a slightly bigger value compared to reported research, and this may be due to the extract used and the synthesis conditions [60,61,62]. The toxicity impact of palladium nanoparticles supported the role of Saudi propolis in anticancer activity on MCF-7 cells, as shown in Figure 14.

## 5. Conclusions

Aqueous Saudi propolis extract and aqueous PdCl_2_ salt solution were used to successfully synthesize palladium nanoparticles. This biosynthesis was controlled by employing optimum conditions (concentrations, ratio of volumes, temperature, pH, and reaction time). A color change from brownish to blackish, followed by UV–visible spectrum analysis, was used to confirm and monitor the development of Pd-NPs. In optimal conditions, the reduction response time was very rapid and completed within 30 min to form Pd-NPs. The flavonoid group resulting in a reduction of Pd^+2^ metal ions was investigated using FTIR analysis. Showing a mean particle size of 4.62 nm, XRD analysis confirmed the crystalline phase and face-centered cubic structure of Pd-NPs. Energy-dispersive X-ray fluorescence spectrometry (EDX) and scanning electron microscopy (SEM) were used to examine the chemical content and the shape of biosynthesized Pd-NPs, which revealed the nanostructure and the impact of Saudi propolis extract. The TEM image revealed an irregular shape with an average particle size of 3.14 nm, which was consistent with the XRD results. Pd-NPs produced by Saudi propolis extract were tested for antibacterial activity against Gram-positive and Gram-negative bacteria and showed a significant zone of inhibition against *Escherichia coli*, *Bacillus subtilis*, and *K. pneumoniae*. Pd-NPs were shown to have excellent antifungal action against *Candida albicans*. The biosynthesized Pd-NPs revealed potent anticancer efficacy against MCF-7 cells with a high IC50 value (104.79 μg/mL). The results of the above studies suggest that biologically produced Pd-NPs may open the path for important medicinal breakthroughs.
